# A novel Gaussian sum quaternion constrained cubature Kalman filter algorithm for GNSS/SINS integrated attitude determination and positioning system

**DOI:** 10.3389/fnbot.2024.1374531

**Published:** 2024-06-07

**Authors:** Qing Dai, Guo-Rui Xiao, Guo-Hua Zhou, Qian-Qian Ye, Shao-Yong Han

**Affiliations:** ^1^College of Urban Construction, Luoyang Polytechnic, Luoyang, China; ^2^Institute of Geospatial Information, Information Engineering University, Zhengzhou, China; ^3^School of Information Engineering and Technology, Changzhou Vocational Institute of Industry Technology, Changzhou, China; ^4^School of Data Science and Artificial Intelligence, Wenzhou University of Technology, Wenzhou, China; ^5^School of Mathematics and Computer Science, Tongling University, Tongling, China; ^6^College of Electrical Engineering, Zhejiang University, Hangzhou, China

**Keywords:** GNSS/SINS integrated attitude determination and positioning system, nonlinear non-Gaussian system, Gaussian mixture model (GMM), Gaussian sum filter algorithm, quaternion cubature Kalman filter algorithm

## Abstract

The quaternion cubature Kalman filter (QCKF) algorithm has emerged as a prominent nonlinear filter algorithm and has found extensive applications in the field of GNSS/SINS integrated attitude determination and positioning system (GNSS/SINS-IADPS) data processing for unmanned aerial vehicles (UAV). However, on one hand, the QCKF algorithm is predicated on the assumption that the random model of filter algorithm, which follows a white Gaussian noise distribution. The noise in actual GNSS/SINS-IADPS is not the white Gaussian noise but rather a ubiquitous non-Gaussian noise. On the other hand, the use of quaternions as state variables is bound by normalization constraints. When applied directly in nonlinear non-Gaussian system without considering normalization constraints, the QCKF algorithm may result in a mismatch phenomenon in the filtering random model, potentially resulting in a decline in estimation accuracy. To address this issue, we propose a novel Gaussian sum quaternion constrained cubature Kalman filter (GSQCCKF) algorithm. This algorithm refines the random model of the QCKF by approximating non-Gaussian noise with a Gaussian mixture model. Meanwhile, to account for quaternion normalization in attitude determination, a two-step projection method is employed to constrain the quaternion, which consequently enhances the filtering estimation accuracy. Simulation and experimental analyses demonstrate that the proposed GSQCCKF algorithm significantly improves accuracy and adaptability in GNSS/SINS-IADPS data processing under non-Gaussian noise conditions for Unmanned Aerial Vehicles (UAVs).

## 1 Introduction

Currently, Global Navigation Satellite Systems (GNSS) and Strapdown Inertial Navigation Systems (SINS) have experienced rapid development in both military and civilian fields. However, a single navigation technology, although it has its advantages in a specific environment, generally performs poorly in conventional occasions and may even fail to complete the task (Groves, 2008; Noureldin et al., 2013). Therefore, combining GNSS and SINS to form a GNSS/SINS integrated positioning and attitude determination system (GNSS/SINS-IADPS) can maximize their advantages and compensate for each other’s limitations (Teunissen and Montenbruck, 2017; Farrell and de Haag, 2020). At present, GNSS/SINS-IADPS has become one of the key technologies in aviation, aerospace and land navigation systems, and receives more attention in military strike, civil aviation, economic construction, and scientific research in various countries (Li and Chen, 2022; Boguspayev et al., 2023). The foundation of GNSS/SINS-IADPS relies on attitude representation methods, which include the attitude quaternion method, Rodrigues parameters and so on (Savage, 1998; Markley, 2003). The attitude quaternion method, in particular, has increasingly garnered attention in the realm of GNSS/SINS-IADPS data processing due to its advantages of avoiding singularities, requiring less computational effort, offering higher accuracy, and enabling complete attitude maneuvers. Consequently, filter algorithms predicated on the attitude quaternion have become a pivotal technology for the processing of GNSS/SINS-IADPS data processing (Ryzhkov, 2021; Michał et al., 2022). However, in practical applications, the mathematical model of GNSS/SINS-IADPS is frequently nonlinear. As such, investigating quaternion-based nonlinear filter algorithms is pivotal for enhancing the efficacy of GNSS/SINS-IADPS data processing (Ali and Mirza, 2010; Zhu et al., 2021).

For a long time in the past, the quaternion extended Kalman filter (QEKF) algorithm has been an important method of quaternion based nonlinear filter algorithm, but this approximately linearized suboptimal filter algorithm has the defect of high-order truncation errors (Gui and de Ruiter, 2018). To overcome the limitations of QEKF algorithm, the quaternion unscented Kalman filter (QUKF) algorithm approximates the probability density distribution of the nonlinear system through the sigma point set, avoiding the linearization errors and solving the Jacobi matrix. Although the filtering estimation performance is improved, frequent switching of quaternions and modification of Rodrigues parameters during iteration result in a large computational burden (Julier et al., 2000; Chang et al., 2013; Challa et al., 2016). The Cubature Kalman filter (CKF) algorithm employs a set of cubature points generated by the third-order sphere-phase diameter cubature rule to approximate the probability density distribution of nonlinear system, which has a more rigorous theoretical basis and better numerical stability than the unscented Kalman Filter algorithm (Arasaratnam et al., 2010; Zhang et al., 2019; Chang et al., 2021). By combining it with the attitude quaternion method, the obtained quaternion cubature Kalman filter (QCKF) algorithm shows the characteristics of simple implementation, good convergence, high precision, and suitability for high-dimensional systems (Geng et al., 2021; Swati, 2022; Wang et al., 2023). Furthermore, the quaternion augmented cubature Kalman filter (QACKF) algorithm enhances the estimation accuracy of QCKF algorithm to some extent, but its computational complexity is significantly higher than that of QCKF algorithm (Wang et al., 2017). For this reason, Huang et al. studied the quaternion state switching cubature Kalman filter (QSSCKF) algorithm, which ensured the filtering estimation accuracy while effectively reducing the computation amount (Huang et al., 2020). However, the use of quaternions as state variables is bound by normalization constraints in practical applications. If these constraints are overlooked during data processing, the resulting accuracy of the filter and the positive quality of covariance will be compromised (Huang et al., 2016; Qiu and Qian, 2018). In addition, these previous studies are based on the assumption that the random model is the white Gaussian noise, and the actual process noise and measurement noise in GNSS/SINS-IADPS deviate from ideal Gaussian distribution. That is to say, GNSS/SINS-IADPS is a nonlinear non-Gaussian system. In such case, if the random model is mismatched, it may affect the accuracy of filtering estimation and even lead to accuracy divergence in severe cases (Duong and Chiang, 2012; Wang et al., 2020; Taghizadeh and Safabakhsh, 2023). Therefore, if the QCKF algorithm can be extended to take into account both the effect of non-Gaussian noise and normalization constraints, it will show better estimation performance.

Researchers have proposed various adaptive filter algorithms to address the problem of non-Gaussian noise in state estimation, which are mainly divided into functional model based adaptive filter and stochastic model based adaptive filter (Mohamed and Schwarz, 1999; Chang, 2014; Elmezayen and El-Rabbany, 2021; Jiang et al., 2021; Yang et al., 2021; Wu et al., 2022). The Sage-Husa filter algorithm can obtain real-time statistical data on current epoch process noise and measurement noise. However, when the moving carrier generates a large disturbance, it is difficult for this kind of filter algorithm to distinguish between the model error and measurement noise, thus affecting the estimation results (Song et al., 2022; Chen et al., 2023). The fading filter algorithm makes the algorithm meet the optimality through a fading factor, but this method is limited to dealing with non-Gaussian process noise only (Sun et al., 2022; Wang et al., 2022). The robust adaptive filter algorithm can handle non-Gaussian noise in both process and measurement noise. When these two types of noise are present, the algorithm can achieve the purpose of stable state estimation results by adjusting the adaptive factor and equivalent weight matrix factor, but this method requires redundant measurement values (Dong et al., 2023). Nonetheless, in the context of the aforementioned research, when dealing with non-Gaussian noise problems, the stochastic model of noise is usually covered by a Gaussian distribution with greater variance.

The Gaussian mixture model (GMM) offers an alternative approach to address the issue of non-Gaussian noise through its multi-mode approximation technique. After being processed by its multi-mode approximation method, it has higher accuracy compared to the traditional extended variance Gaussian distribution approximation method (Alspach and Sorenson, 1972). In recent years, the filter random model has been optimized by GMM, which has gradually been recognized as a superior approach, attracting attention in target tracking, speech recognition, signal analysis, navigation integrity monitoring, and other aspects (Sun et al., 2020; Zickert and Yarman, 2021; Zhu et al., 2022; Yu et al., 2023). The literature sequentially delves into the nonlinear optimization problem associated with the Gaussian sum filter (GSF) algorithm grounded in GMM, such as the Gaussian sum extended Kalman filter (GSEKF) algorithm, Gaussian sum unscented Kalman filter (GSUKF) algorithm, Gaussian sum quadrature Kalman Filter (GSQKF) algorithm, and Gaussian sum cubature Kalman filter (GSCKF) algorithm, and these studies have to some extent optimized the integrated navigation information fusion algorithm (Wang and Cheng, 2015; Qian et al., 2021; Wang et al., 2021; Bai et al., 2022). While numerous algorithms have been put forward to optimize the random model of nonlinear filter algorithms under non-Gaussian noise conditions based on GMM, there are limited reports on research on quaternion-based algorithm in GNSS/SINS-IADPS data processing for UAVs.

To tackle the issue of random model mismatch under non-Gaussian noise conditions and quaternions normalization constraints affecting the QCKF algorithm, which leads to degradation in estimation accuracy during GNSS/SINS-IADPS data processing for UAVs, a novel Gaussian sum quaternion constrained cubature Kalman filter (GSQCCKF) algorithm is proposed in this paper. The algorithm combines the GMM principle with the two-step projection method and improves the QCKF algorithm. Firstly, multiple sub-filters are decomposed using GMM. Secondly, the quaternion is restricted by the two-step projection method to achieve the purpose of quaternion normalization in attitude determination. Finally, simulation and experiments in GNSS/SINS-IADPS data processing for UAVs are conducted to verify the improvement of estimation accuracy and adaptability for GSQCCKF. The outcomes demonstrate that the proposed GSQCCKF algorithm significantly mitigates the adverse effects of non-Gaussian noise on state estimation, substantially improving both accuracy and adaptability in the GNSS/SINS-IADPS data processing utilized on UAVs.

## 2 Preliminaries and problem formulation

### 2.1 Mathematical models for GNSS/SINS-IADPS

We have adopted an integrated navigation system composed of single antenna GNSS and SINS, which is tightly integrated in combination. This tightly integrated GNSS/SINS-IADPS has better navigation accuracy and anti-interference performance than the individual attitude determination and positioning technology of GNSS and SINS, so it is widely used in numerous scientific fields. In the GNSS/SINS-IADPS, the QEKF algorithm is generally used to fuse GNSS and SINS navigation information. To use the QEKF algorithm, it is customary to use the linearized GNSS/SINS-IADPS mathematical model. However, when the GNSS/SINS-IADPS works in high maneuverability, it will show obvious nonlinear characteristics. At this time, if the GNSS/SINS-IADPS mathematical model is linearized, the estimation accuracy will be reduced because of the linearization error. Therefore, in order to cope with linearization errors, need to establish the nonlinear mathematical model of GNSS/SINS-IADPS. The nonlinear mathematical models consist of two components: state-space equation and measurement equation.

The state estimate of GNSS/SINS-IADPS at epoch k−1 is defined in formula (1).


(1) $$x_{k-1|k-1}=\left[\delta q,\delta v,\delta p,\varepsilon ,\nabla \right]$$

here, δq is attitude quaternion errors, δv is velocity errors, δp is position errors, ε is the gyroscope biases drift, ∇ is the accelerometer biases drift. The state-space equation of GNSS/SINS-IADPS is described as follows:


(2) $$x_{k|k-1}=f\left(x_{k-1|k-1}\right)+g_{k}w_{k}$$

here, $x_{k|k-1}$ is the state prediction, f(⋅) is a nonlinear function, $g_{k}$ is the system noise driven matrix, $w_{k}$ is the process noise. Assuming that $w_{k}$ is characterized as white Gaussian noise, it can be represented mathematically as $w_{k}\sim \left(0,Q_{k}\right)$ .

The measurement $z_{k}$ of the measurement equation is composed of the corrected pseudo-range and pseudo-range rate, which have been adjusted for the satellite clock bias, tropospheric delay, and ionospheric delay. Then, according to the state prediction $x_{k|k-1}$ determined in formula (2) and measurement $z_{k}$ , the measurement equation of GNSS/SINS-IADPS is established:


(3) $$z_{k}=h\left(x_{k|k-1}\right)+v_{k}$$

here, h(⋅) is a nonlinear measurement function; $v_{k}$ is the measurement noise, which is caused by receiver noise, multipath effects, and orbit prediction errors. Assuming that the measurement noise $v_{k}$ is characterized as white Gaussian noise, it can be represented mathematically as $v_{k}\sim \left(0,R_{k}\right).$

### 2.2 Quaternion cubature Kalman filter algorithm

The QCKF algorithm is a Gaussian filter algorithm that estimates the posterior distribution of the probability density function (PDF) of a nonlinear function by utilizing a set of cubature points, thereby circumventing the necessity for linearization of the nonlinear function (Geng et al., 2021; Swati, 2022; Wang et al., 2023). The concrete implementation procedures of the QCKF algorithm are outlined below:

Step 1: The selection of the cubature points $\chi_{c,k-1|k-1}$ is accomplished through the utilization of the third-order sphere-phase diameter cubature rule in formulas (4, 5).


(4) $$\chi_{c,k-1|k-1}=S_{k-1|k-1}\xi_{c}+x_{k-1|k-1}$$

(5) $$\xi_{c}=\sqrt{\frac{m}{2}}{\left\{\left(\begin{array}{c} 1\\ \vdots \\ 0 \end{array}\right),\cdots ,\left(\begin{array}{c} 0\\ \vdots \\ 1 \end{array}\right),\left(\begin{array}{c} -1\\ \vdots \\ 0 \end{array}\right),\cdots ,\left(\begin{array}{c} 0\\ \vdots \\ -1 \end{array}\right)\right\}}_{i}$$

here, the Cholesky decomposition of the matrix $S_{k-1|k-1}$ can be obtained as $P_{k-1|k-1}=S_{k-1|k-1}S_{k-1|k-1}^{T}$ , $P_{k-1|k-1}$ is the covariance pertains to the state estimation at epoch k−1 , and c=1,2,⋯,m=2n , n is the dimension of the state estimation, that is, the overall quantity of cubature points is double the dimensionality of the state estimation.

Step 2: Prediction.

The propagated cubature points $x_{c,k|k-1}^{*}$ are estimated through the nonlinear function f(⋅) , which can be expressed in formula (6).


(6) $$x_{c,k|k-1}^{*}=f\left(\chi_{c,k-1|k-1}\right)$$

Then, the state prediction $x_{k|k-1}$ at epoch k can be computed, as illustrated in formula (7).


(7) $$x_{k|k-1}=\frac{1}{m}\overset{m}{\underset{c=1}{\sum }}x_{c,k|k-1}^{*}$$

The state prediction covariance $P_{k|k-1}$ can be derived, as depicted in formula (8).


(8) $$P_{k|k-1}=\frac{1}{m}\overset{m}{\underset{c=1}{\sum }}x_{c,k|k-1}^{*}x_{c,k|k-1}^{*T}-x_{k|k-1}x_{k|k-1}^{T}+Q_{k}$$

Step 3: Update.

The cubature points $\chi_{c,k|k-1}$ can be re-estimated in formula (9).


(9) $$\chi_{c,k|k-1}=S_{k|k-1}\xi_{c}+x_{k|k-1}$$

here, $P_{k|k-1}=S_{k|k-1}S_{k|k-1}^{T}$ .

The propagated cubature points $z_{c,k|}^{*}{\;}_{k-1}$ can be estimated by applying the nonlinear function h(⋅) , which is expressed in formula (10).


(10) $$z_{c,k|}^{*}{\;}_{k-1}=h\left(\chi_{c,k|k-1}\right)$$

Subsequently, the measurement prediction $z_{k|k-1}$ can be computed, as illustrated in formula (11).


(11) $$z_{k|k-1}=\frac{1}{m}\overset{m}{\underset{c=1}{\sum }}z_{c,k|}^{*}{\;}_{k-1}$$

The covariance $P_{zz,k|k-1}$ of the measurement prediction, cross-covariance $P_{xz,k|k-1}$ of the measurement prediction, and the filter gain $K_{k}$ can be derived, as depicted in formulas (12)–(14), respectively.


(12) $$P_{zz,k|k-1}=\frac{1}{m}\overset{m}{\underset{c=1}{\sum }}z_{c,k|}^{*}{\;}_{k-1}z_{c,k|}^{*T}{\;}_{k-1}-z_{k|k-1}z_{k|k-1}^{T}+R_{k}$$

(13) $$P_{xz,k|k-1}=\frac{1}{m}\overset{m}{\underset{c=1}{\sum }}\chi_{c,k|k-1}z_{c,k|}^{*T}{\;}_{k-1}-x_{k|k-1}z_{k|k-1}^{T}$$

(14) $$K_{k}=P_{xz,k|k-1}P_{zz,k|k-1}^{-1}$$

Subsequently, the state estimation $x_{k|k}$ and its covariance $P_{k|k}$ at epoch k can be obtained, which are expressed in formulas (15) and (16).


(15) $$x_{k|k}=x_{k|k-1}+K_{k}\left(z_{k}-z_{k|k-1}\right)$$

(16) $$P_{k|k}=P_{k|k-1}-K_{k}P_{zz,k|k-1}K_{k}^{T}$$

### 2.3 The limitation of QCKF algorithm

The wavelet transform method is employed to analyze the statistical properties of SINS errors, and Allan variance analysis is utilized to scrutinize the statistical characteristics of GNSS residuals (El-Sheimy et al., 2008; Wang et al., 2018; Zhang et al., 2020). From the analysis results, it was found that SINS errors and GNSS residuals do not conform to the distribution of zero-mean white Gaussian noise. Instead, the SINS errors and GNSS residuals remnants are a mixed distribution of Gaussian noise and non-Gaussian noise (El-Sheimy et al., 2008; Wang et al., 2018; Zhang et al., 2020). Since the QCKF algorithm is based on the white Gaussian noise hypothesis, this hypothesis may lead to suboptimal estimation results for GNSS/SINS-IADPS due to the random model mismatch inherent in the QCKF algorithm.

In addition, the quaternion normalization problem exists in the attitude quaternion of state estimation. Neglecting the quaternion constraint during filtering calculations may result in a decline in the estimation accuracy, potentially leading to covariance singularity.

Therefore, to enhance the QCKF algorithm estimation performance of the GNSS/SINS-IADPS under non-Gaussian noise environments, it is necessary to further refine the random model of QCKF algorithm and optimize its algorithm model employed in the GNSS/SINS-IADPS.

## 3 Gaussian sum quaternion constrained cubature Kalman filter algorithm

The QCKF algorithm based on the assumption of white Gaussian noise is difficult to obtain ideal performance in state estimation due to the influence of random model mismatch and the oversight of quaternion normalization. In this section, a novel GSQCCKF algorithm is proposed, which is based on the QCKF algorithm framework. The aim of this algorithm is to address the state estimation problem of QCKF algorithm used in non-Gaussian noise environment for GNSS/SINS-IADPS data processing. The steps involved in the GSQCCKF algorithm are described as follows.

### 3.1 Modeling of non-Gaussian probability density function by GMM

Non-Gaussian noise can be modeled as a multi-component system based on the degree of nonlinearity or the maximum eigenvalue of the covariance matrix (Maebashi et al., 2008; Liu et al., 2014; Qian et al., 2021). The PDF of $x_{k-1|k-1}$ in formula (1) can be approximately expressed as follows:


(17) $$\begin{array}{l} p\left(x_{k-1|k-1}\right)\approx \overset{I}{\underset{i=1}{\sum }}\omega_{k-1}\left(i\right)N\left(\begin{array}{l} x_{k-1|k-1};{\bar{\mu }}_{k-1}\left(i\right),\\ P_{k-1|k-1}\left(i\right) \end{array}\right)\;,\;\\ \overset{I}{\underset{i=1}{\sum }}\omega_{k-1}\left(i\right)=1 \end{array}$$

here, $\omega_{k-1}\left(i\right)$ denotes the weight of the ith Gaussian component, $N\left(x_{k-1|k-1};{\bar{\mu }}_{k-1}\left(i\right),P_{k-1|k-1}\left(i\right)\right)$ represents the ith Gaussian component with a mean of ${\bar{\mu }}_{k-1}\left(i\right)$ and a variance of $P_{k-1|k-1}\left(i\right)$ , I signifies the total number of Gaussian components.

Correspondingly, the PDF of the process noise $p\left(w_{k}\right)$ in formula (2) and the PDF of the measurement noise $p\left(v_{k}\right)$ in formula (3) can be approximately expressed as follows:


(18) $$p\left(w_{k}\right)\approx \overset{J}{\underset{j=1}{\sum }}\alpha_{k}\left(j\right)N\left(w_{k};{\bar{w}}_{k}\left(j\right),Q_{k}\left(j\right)\right)\;,\;\overset{J}{\underset{i=1}{\sum }}\alpha_{k}\left(j\right)=1$$

(19) $$p\left(v_{k}\right)\approx \overset{L}{\underset{l=1}{\sum }}\beta_{k}\left(l\right)N\left(v_{k};{\bar{v}}_{k}\left(l\right),R_{k}\left(l\right)\right)\;,\;\overset{L}{\underset{l=1}{\sum }}\;\beta_{k}\left(l\right)=1$$

here, $\alpha_{k}\left(j\right)$ represents the weight of the jth Gaussian component, $N\left(w_{k};{\bar{w}}_{k}\left(j\right),Q_{k}\left(j\right)\right)$ represents the jth Gaussian component with a mean of ${\bar{w}}_{k}\left(j\right)$ and a variance of $Q_{k}\left(j\right)$ , J represents the total number of Gaussian components; $\beta_{k}\left(l\right)$ represents the weight of the lth Gaussian component, $N\left(v_{k};{\bar{v}}_{k}\left(l\right),R_{k}\left(l\right)\right)$ represents the lth Gaussian component with a mean of ${\bar{v}}_{k}\left(l\right)$ and a variance of $R_{k}\left(l\right)$ , L represents the total number of Gaussian components.

### 3.2 Gaussian sum quaternion cubature Kalman filter algorithm

Following the implementation of the non-Gaussian PDF through GMM, the prediction and update on each sub-filters are carried out.

Step 1: Prediction.

The cubature points $\chi_{c,k-1|k-1}$ are formulated utilizing the third-order sphere-phase diameter cubature rule.


(20) $$\chi_{c,k-1|k-1}\left(i\right)=S_{k-1|k-1}\left(r\right)\cdot \xi_{c}+x_{k-1|k-1}\left(i\right)$$

here, $S_{k-1|k-1}\left(r\right)$ is derived through Cholesky decomposition of $P_{k-1|k-1}\left(r\right)$ , which can be expressed as $P_{k-1|k-1}\left(r\right)=S_{k-1|k-1}\left(r\right)S_{k-1|k-1}^{T}\left(r\right)$ , r=(i−1)⋅I+j .

By propagating the cubature points $\chi_{c,k-1|k-1}\left(i\right)$ through the nonlinear function f(⋅) , we can obtain $\chi_{c,k|k-1}^{*}\left(i\right)=f\left(\chi_{c,k-1|k-1}\left(i\right)\right).$ The state prediction $x_{k|k-1}\left(r\right)$ is calculated as follows:


(21) $$x_{k|k-1}\left(r\right)=\overset{m}{\underset{c=1}{\sum }}\omega_{c}\chi_{c,k|k-1}^{*}\left(i\right)+{\bar{w}}_{k}\left(j\right)$$

Subsequently, the covariance of state prediction $P_{k|k-1}\left(r\right)$ can be derived, as depicted in formula (22).


(22) $$\begin{array}{l} P_{k|k-1}\left(r\right)=\overset{m}{\underset{c=1}{\sum }}\omega_{c}\chi_{c,k|k-1}^{*}\left(i\right)\chi_{c,k|k-1}^{*}{\;}^{T}\left(i\right)-\\ {}[x_{k|k-1}\left(r\right)-\;{\bar{w}}_{k}\left(j\right)]\\ {\left[x_{k|k-1}\left(r\right)-{\bar{w}}_{k}\left(j\right)\right]}^{T}+Q_{k}\left(j\right)\; \end{array}$$

here, $\omega_{c}$ denotes the weight of the cubature points.

Step 2: Update.

The cubature points are assessed:


(23) $$\chi_{c,k|k-1}\left(r\right)=S_{k|k-1}\left(r\right)\xi_{c}+x_{k|k-1}\left(r\right)$$

Here,


$$P_{k|k-1}\left(r\right)=S_{k|k-1}\left(r\right)S_{k|k-1}^{T}\left(r\right).$$

By propagating the cubature points $\chi_{c,k|k-1}\left(r\right)$ through the nonlinear function h(⋅) , the propagated cubature points can be obtained as: $z_{c,k|k-1}^{*}\left(r\right)=h\left(\chi_{c,k|k-1}\left(r\right)\right).$ The measurement prediction $z_{k|k-1}\left(r,l\right)$ is calculated as follows:


(24) $$z_{k|k-1}\left(r,l\right)=\overset{m}{\underset{c=1}{\sum }}\omega_{c}z_{c,k|k-1}^{*}\left(r\right)+{\bar{v}}_{k}\left(l\right)$$

The covariance matrix of measurement prediction $P_{zz,k|k-1}\left(r,l\right)$ , the cross-covariance $P_{xz,k|k-1}\left(r,l\right)$ , the filter gain $K_{k}\left(r,l\right)$ , the state estimation $x_{k|k}\left(g\right)$ and its corresponding covariance $P_{k|k}\left(g\right)$ can be derived, as depicted in formulas (25)–(29), respectively.


(25) $$\begin{array}{l} P_{zz,k|k-1}\left(r,l\right)=\overset{m}{\underset{c=1}{\sum }}\omega_{c}\chi_{c,k|k-1}\left(r\right)\chi_{c,k|k-1}^{T}\left(r\right)-\\ \left(z_{k|k-1}\left(r,l\right)-\bar{v}\left(l\right)k\;\right)\\ {\left(\begin{array}{l} z_{k|k-1}\left(r,l\right)-\\ \bar{v}\left(l\right)k\; \end{array}\right)}^{T}+R_{k}\left(l\right)\; \end{array}$$

(26) $$\begin{array}{l} P_{xz,k|k-1}\left(r,l\right)=\overset{m}{\underset{c=1}{\sum }}\omega_{c}\chi_{c,k|k-1}^{*}\left(r\right)z_{c,k|k-1}^{*}{\left(r\right)}^{T}-\\ x_{k|k-1}\left(r\right){\left[z_{k|k-1}\left(r,l\right)-\bar{v}\left(l\right)k\;\right]}^{T}\; \end{array}$$

(27) $$K_{k}\left(r,l\right)=P_{xz,k|k-1}\left(r,l\right)P_{zz,k|k-1}^{-1}\left(r,l\right)$$

(28) $$x_{k|k}\left(g\right)=x_{k|k-1}\left(r\right)+K_{k}\left(r,l\right)\left[z_{k}-z_{k|k-1}\left(r,l\right)\right]$$

(29) $$P_{k|k}\left(g\right)=P_{k|k-1}\left(r\right)-K_{k}\left(r,l\right)P_{zz,k|k-1}\left(r.l\right)K_{k}^{T}\left(r,l\right)$$

Here,


g=(r−1)L+l.

Step 3: Global point estimate.

The state estimation $x_{k|k}$ and its covariance matrix $P_{k|k}$ are computed as


(30) $$x_{k|k}=\overset{I\cdot J\cdot L}{\underset{g=1}{\sum }}\omega_{k}\left(g\right)x_{k|k}\left(g\right)$$

(31) $$\begin{array}{l} P_{k|k}=\overset{I\cdot J\cdot L}{\underset{g=1}{\sum }}\omega_{k}\left(g\right)(P_{k|k}\left(g\right)+\\ \left(x_{k|k}\left(g\right)-x_{k|k}\right){\left(x_{k|k}\left(g\right)-x_{k|k}\right)}^{T})\; \end{array}$$

here, $\omega_{k}\left(g\right)$ denotes the weight of the gth Gaussian component, which is calculated as formula (32).


(32) $$\omega_{k}\left(g\right)=\frac{\alpha_{k|k-1}\left(r\right)\beta_{k}\left(l\right)p\left(z_{k|k}|x_{k|k},g\right)}{\overset{I\cdot J}{\underset{r=1}{\sum }}\overset{L}{\underset{l=1}{\sum }}\alpha_{k|k-1}\left(r\right)\beta_{k}\left(l\right)p\left(z_{k|k}|x_{k|k},g\right)}$$

In this regard, the PDF of the gth Gaussian component is given by, and its computation can be expressed as follows:


(33) $$p\left(z_{k}|x_{k|k},g\right)=\frac{1}{\sqrt{2\pi \sigma_{g}^{2}}}\exp\left(-\frac{1}{2}{\left(\frac{z_{k}-z\left(r,l\right)k|k-1\;}{\sigma_{g}}\right)}^{2}\right)$$

From formulas (32) and (33), it is evident that the weight $\omega_{k}\left(g\right)$ of the Gaussian component adaptively modifies in response to the innovation $z_{k}-z\left(r,l\right)k|k-1\;$ , thereby enhancing the robustness of the filter algorithm.

Step 4: Gaussian component reduction.

From formula (20) to (30), it can be found that after step 3 the number of Gaussian components reaches I⋅J⋅L . If I⋅J⋅L>I , there will be a mismatch between the number of Gaussian components at epoch k and epoch k − 1 when the recurrence operation is performed at epoch k. It can be seen that the total number of Gaussian components will increase with each iteration of filtering, which will eventually lead to an exponential increase in filtering recursion. Therefore, it is necessary to reduce the number of Gaussian components after each iteration, this ensures that the total number of Gaussian components in the state estimation remains I . Gaussian component merging generally employs a quasi-Bayesian approximation, but the threshold selection depends on experience. Here, we adopt a different approach, by arranging the weight values of the Gaussian components in descending order, the Gaussian components are then sequentially labeled as g=1,⋯,I⋅J⋅L , while retaining I−1 components, then the weight value $\omega_{k}\left(I\right)$ of the I−1 Gaussian component at epoch k can be calculated:


(34) $$\omega_{k}\left(I\right)=\overset{J\cdot L}{\underset{g=1}{\sum }}\omega_{k}\left(g\right)$$

The mean of state estimation $x_{k|k}\left(I\right)$ and its covariance $P_{k|k}\left(I\right)$ corresponding to the Ith Gaussian component is shown in formulas (35) and (36), respectively.


(35) $$x_{k|k}\left(I\right)={\overset{J\cdot L}{\underset{n=1}{\sum }}}_{k}\tilde{\omega }\left(g\right)x_{k|k}\left(g\right)$$

(36) $$\begin{array}{l} P_{k|k}\left(I\right)=\overset{J\cdot L}{\underset{n=1}{\sum }}{\tilde{\omega }}_{k}\left(g\right)[P\left(g\right)k|k\;+\left(x_{k|k}\left(g\right)-x_{k|k}\left(I\right)\right)\;\\ {\left(x_{k|k}\left(g\right)-x_{k|k}\left(I\right)\right)}^{T}]\;\;\; \end{array}$$

here, ${\tilde{\omega }}_{k}\left(g\right)=\omega_{k}\left(g\right)/\omega_{k}\left(I\right)$ denotes the regularization weight. Through the aforementioned procedures, I−1 Gaussian components and the Ith Gaussian component as described in formulas (35) and (36) are employed for the filtering recursion in the subsequent epoch. It is evident that following the merging of Gaussian components, the overall number of Gaussian components within the filter algorithm remains unchanged.

### 3.3 Two-step projection method

The two-step projection method (Tang et al., 2012; Huang, 2017) is employed herein to address quaternion constraint problem. In the initial step, the unconstrained state estimation distribution is projected onto the constrained surface so that the attitude estimation distribution complies with the quaternion constraint. However, this action may result in a reduction in the variance of attitude estimation. In the subsequent step, the constrained state estimation distribution is projected onto the constrained surface so that the mean of the attitude estimation satisfies the quaternion constraint. Simultaneously, the attitude estimation variance is compensated, to enhance the accuracy of attitude estimation while ensuring quaternion normalization. The specific processing steps are outlined as follows:

Step 1: the mean of constrained state estimation distribution ${\vec{x}}_{k|k}$ and its covariance ${\vec{P}}_{k|k}$ can be computed, which are detailed below:


(37) $${\vec{x}}_{k|k}=\overset{2n}{\underset{c=1}{\sum }}\omega_{c}{\tilde{\chi }}_{c,k|k}$$

(38) $${\vec{P}}_{k|k}=\overset{2n}{\underset{c=1}{\sum }}\omega_{c}\left({\tilde{\chi }}_{c,k|k}-{\vec{x}}_{k|k}\right){\left({\tilde{\chi }}_{c,k|k}-{\vec{x}}_{k|k}\right)}^{T}$$

here, ${\tilde{\chi }}_{c,k|k}=\varphi \left(\chi_{c,k|k}^{*}\right),$ $\chi_{c,k|k}^{*}={\bar{S}}_{k|k}\xi_{c}+x_{k|k},$ $P_{k|k}={\bar{S}}_{k|k}{\bar{S}}_{k|k}^{T}$ , φ(⋅) is the constraint function.

Step 2: the ultimate state estimation ${\tilde{x}}_{k|k}$ and its covariance ${\tilde{P}}_{k|k}$ can be calculated:


(39) $${\tilde{x}}_{k|k}=\varphi \left({\vec{x}}_{k|k}\right)$$

(40) $${\tilde{P}}_{k|k}={\vec{P}}_{k|k}+\left({\tilde{x}}_{k|k}-{\vec{x}}_{k|k}\right){\left({\tilde{x}}_{k|k}-{\vec{x}}_{k|k}\right)}^{T}$$

### 3.4 GSQCCKF algorithm structure

**Figure 1:**
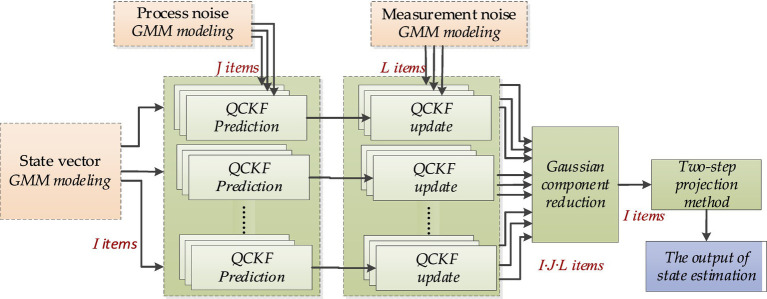
The flow diagram of proposed GSQCCKF algorithm.

The GSQCCKF algorithm is implemented in five stages, as illustrated in Figure 1.

Stage 1: State estimation, process noise, and measurement noise are modeled by GMM, as shown in formulas (17)–(19).

Stage 2: The prediction of GSQCKF algorithm at epoch k is calculated, as shown in formulas (20)–(22).

Stage 3: The Update of GSQCKF algorithm at epoch k is calculated, as shown in formulas (23)–(29).

Stage 4: Global point estimate (formulas 30–33) and then perform Gaussian component reduction (formulas 34–36).

Stage 5: The two-step projection method is applied to deal with the quaternion normalization issue, and the output of state estimation at epoch k , as shown in formulas (39) and (40).

## 4 Performance evaluation and discussion

In this section, the performance of the GSQCCKF algorithm was assessed, then, a comparative analysis was conducted on the performance of four different algorithms (GSQCCKF, QEKF, QCKF, and QCCKF).

### 4.1 Simulations and analysis

**Figure 2:**
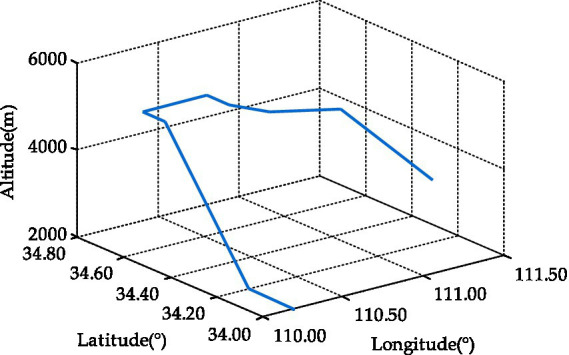
The UAV flight trajectory.

The performance of proposed GSQCCKF algorithm was evaluated by the Monte Carlo simulations, which were based on the design of a dynamic UAV equipped with a GNSS/SINS-IADPS. The flight trajectory of the UAV is depicted in Figure 2, which includes a variety of maneuvering states, such as climbing, level flight, turning and descending. The initial attitude of UAV was set as roll 0°, pitch 0°, and yaw 0°, while its initial speed was specified as 0 m/s in East, 120 m/s in North, and 0 m/s in Up. The initial position of UAV was established at 110.2° longitude, 34.0° latitude, and 2000 m altitude. Additionally, the initial attitude error of UAV was specified as roll 1 “, pitch 1 “, yaw 1.5 “, while its initial velocity error was set at 0.3 m/s in the East, 0.3 m /s in the North, 0.3 m/s in the Up direction, and the initial position error of UAV was defined as 10 m longitude, 10 m latitude, and 10 m altitude. The SINS parameter configurations of the GNSS/SINS-IADPS are presented in Table 1. The GNSS measurement was simulated based on the satellite constellation and epoch information on June 13, 2023. The pseudo range observation error of the GNSS receiver was 10m , and the sampling frequency of the GNSS receiver was 1 Hz.

**Table 1:** Parameters of SINS.

Parameter	Value
Constant drift of gyroscope	0.1(o/h)
Random walk coefficient of gyroscope	$0.01\left(o/\sqrt{h}\right)$
Zero bias of accelerometer	0.001(g)
Random walk coefficient of accelerometer	$0.0001\;\left(g\cdot \sqrt{s}\right)$
Sampling rate	50(Hz)

White Gaussian noise scenario and non-Gaussian noise scenario were simulated separately. For each scenario, 100 Monte Carlo simulations were carried out, and each simulation time was 1,500 s. The configuration of the computing platform used in the simulation is as follows. CPU: Inter Core i7-12700, 2.9GHZ; Internal memory: DDR4 16GB; Simulation software: Matlab R2020b. The precision of each filter estimation was assessed using the root mean square error (RMSE), which is mathematically expressed in formula (41).


(41) $$\mathrm{RMSE}=\sqrt{\frac{1}{N}\overset{N}{\underset{i=1}{\sum }}{\left({\bar{X}}_{i}-X_{i}\right)}^{2}}$$

here, N signifies the total number of Monte Carlo simulations; $X_{i}$ represents the reference; ${\bar{X}}_{i}$ signifies the estimation.

#### 4.1.1 White Gaussian noise scenario

In this scenario, both process noise and observation noise are simulated as white Gaussian noise, the covariance of which are defined in formula (42).


(42) $$Q=diag\left[\begin{array}{l} {\left(0.01^{\circ }/\sqrt{h}\right)}^{2}I_{3\times 3},{\left(1\times 0.0001g\cdot \sqrt{s}\right)}^{2}\\ I_{3\times 3},0_{9\times 9},10m/\sqrt{s},\;5m/s^{3/2} \end{array}\right]$$

$$R={\left(25m\right)}^{2}I_{4\times 4}$$

**Figure 3:**
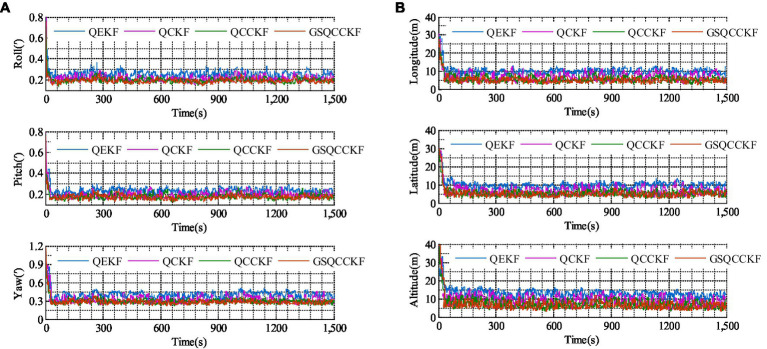
RMSEs of the attitude and position obtained by four different algorithms for the white Gaussian noise scenario. **(A)** Attitude. **(B)** Position.

The RMSE of attitude and position results calculated by four different algorithms (QEKF, QCKF, QCCKF, and GSQCCKF) under white Gaussian noise scenario are shown in Figure 3, where the epoch range ranges from 0 s to 1500s.

**Figure 4:**
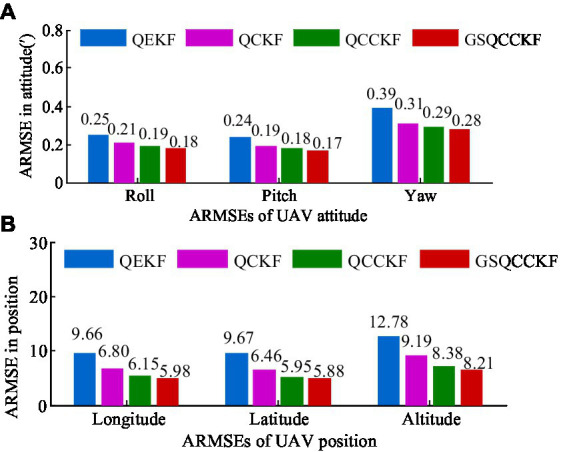
ARMSEs of the position and attitude obtained by four different algorithms for the white Gaussian noise scenario. **(A)** Attitude. **(B)** Position.

Additionally, the average RMSEs (ARMSEs) for each algorithm are also computed and presented in Figure 4 for ease of comparison.

As is evident from Figures 3, 4, QEKF, QCKF, QCCKF, and GSQCCKF, these four algorithms all demonstrate convergence in the white Gaussian noise scenario. Our analysis reveals that the QEKF algorithm exhibits the highest estimation error. This can be attributed to the fact that when the initial attitude error is present in the QEKF algorithm, the high-order truncation error within the filter leads to a reduction in estimation accuracy and filtering stability. In contrast, algorithms of QCKF, QCCKF, and GSQCCKF are all derived from the QCKF algorithm framework. The QCKF algorithm computes the state estimation and its covariance using a set of cubature points, thus enabling algorithms of QCKF, QCCKF, and GSQCCKF to mitigate the impact of nonlinear function linearization errors inherent in the QEKF algorithm on estimation accuracy.

In comparison to the QCKF algorithm, both the QCCKF algorithm and the GSQCCKF algorithm exhibit a marked enhancement in estimation accuracy. This is because both of them consider the quaternion constraint in the state estimation, so the filter gain calculated by these two algorithms is also constrained, which further improves the estimation accuracy of attitude and position. It is noteworthy that the computational accuracy of the QCCKF algorithm and the GSQCCKF algorithm are similar to each other. This outcome is attributable to the fact that the QCCKF algorithm is based on the assumption of white Gaussian noise, while the GSQCCKF represents an enhancement of the QCCKF, grounded in the GMM approach. In white Gaussian noise scenario, both the QCCKF algorithm and the GSQCCKF algorithm are capable of converging, resulting in similar estimation accuracies.

#### 4.1.2 Non-Gaussian noise scenario

**Figure 5:**
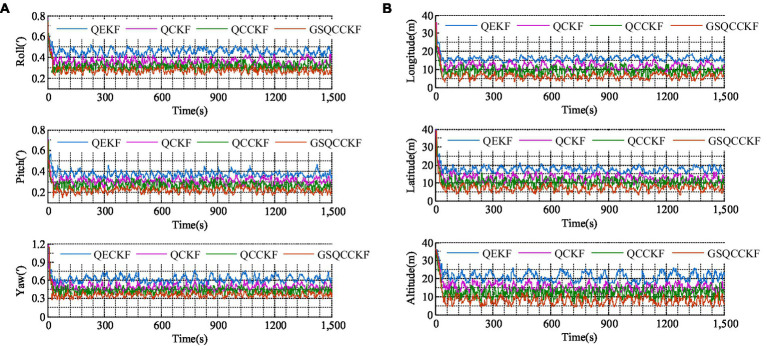
RMSEs of the attitude and position obtained by four different algorithms for the non-Gaussian noise scenario. **(A)** Attitude. **(B)** Position.

**Figure 6:**
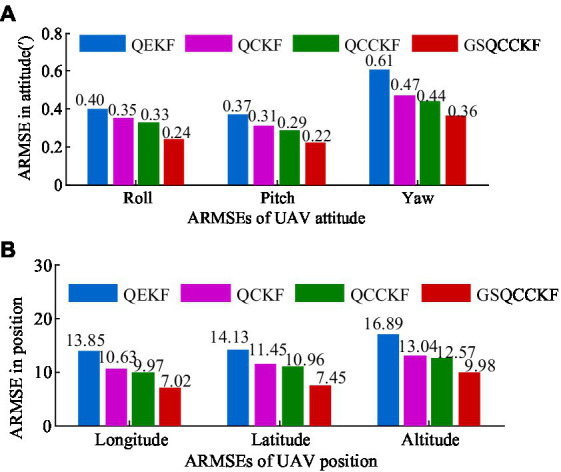
ARMSEs of the attitude and position obtained by four different algorithms for the non-Gaussian noise scenario. **(A)** Attitude. **(B)** Position.

In non-Gaussian noise scenario, non-Gaussian process noise is modeled and generated by the model $0.9N\left(0,Q_{k}\right)+\left(1-0.9\right)N\left(0,10Q_{k}\right),$ while the non-Gaussian measurement noise is also modeled and generated by the model $0.9N\left(0,R_{k}\right)+\left(1-0.9\right)N\left(0,10R_{k}\right).$ The RMSEs of attitude and position which result from four different algorithms (QEKF, QCKF, QCCKF, and GSQCCKF) are shown in Figure 5 and their corresponding ARMSEs are illustrated in Figure 6, where the epoch range ranges from 0 s to 1500s.

As can be observed from Figures 5, 6, the estimation errors of QEKF algorithm, QCKF algorithm, and QCCKF algorithm increase significantly in the non-Gaussian noise scenario when compared to the white Gaussian noise scenario. Taking the yaw estimation results as example, the yaw ARMSEs calculated by algorithms of QEKF, QCKF, and QCCKF increased from 0.39′, 0.31′, 0.29′ to 0.61′, 0.47′, 0.44′, with error increase rates approximately 56.41, 52.61, and 51.72%, respectively. In contrast, the variation of the estimation error of GSQCCKF algorithm is the smallest. And the ARMSE calculated by the GSQCCKF algorithm changes from 0.28′ to 0.36′, resulting in an error increase rate of about 28.57%. It indicates that the GSQCCKF algorithm exhibits the highest estimation accuracy in the presence of non-Gaussian noise. The reason for this phenomenon lies in the fact that the GSQCCKF algorithm enhances the filtering estimation accuracy by refining the random model through GMM, and this optimization method can make the adaptability of the algorithm more effectively to mitigate the impact of non-Gaussian noise on the GNSS/SINS-IADPS data processing.

#### 4.1.3 Computational performance

**Figure 7:**
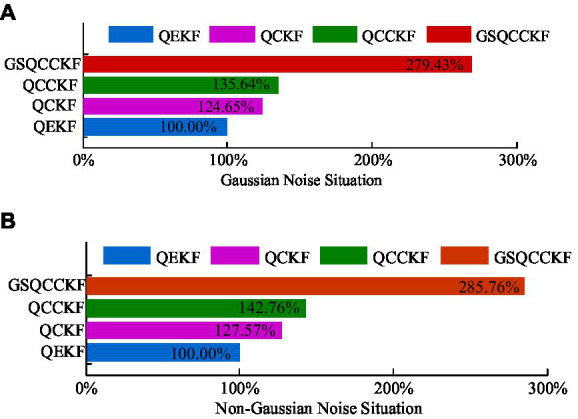
Relative computational efficiencies of four different algorithms. **(A)** White Gaussian noise scenario. **(B)** Non-Gaussian noise scenario.

The computational time per epoch run, for four different algorithms (QEKF, QCKF, QCCKF, and GSQCCKF), as shown in Table 2. In addition, the computational efficiency of them was compared in Figure 7.

**Table 2:** Computational time per epoch run of four different algorithms.

Algorithm	The average time spent per epoch (ms)	
	White Gaussian noise scenario	Non-Gaussian noise scenario
QEKF	5.42	5.75
QCKF	6.76	7.34
QCCKF	7.35	8.21
GSQCCKF	15.15	16.43

As depicted in Table 2 and Figure 7, it reveals that the changes of computation time for all these four algorithms are relatively similar in white Gaussian noise scenario and non-Gaussian noise scenario. The QEKF algorithm exhibits the shortest computational time. However, the computational time of QCKF algorithm is at least 27.57% longer than that of QEKF algorithm, due to the complex and time-consuming process involved in cubature transformation calculations. Further, the computational time of the QCCKF algorithm is at least 15.19% larger than that of the QCKF algorithm owing to the calculation of the quaternion constraint. Due to the complexity of the computational process, the GSQCCKF algorithm takes the longest computational time, approximately twice that of QCCKF algorithm, as it performs distributed filtering and global point estimation at each iteration. Fortunately, the Gaussian components are trimmed and merged during the processing, preventing exponential growth in computational time (accounting for approximately 285.76% of QEKF). Furthermore, with the significant increase in computing power today, the millisecond-level operation time of GSQCCKF algorithm (16.43 ms) can still meet the real-time data processing requirements of GNSS/SINS-IADPS. In conclusion, despite its increased computational time, the GSQCCKF algorithm remains capable of handling high-dynamic navigation for GNSS/SINS-IADPS equipped on UAVs.

The simulation analysis and comparison conducted in sections 4.1.1, 4.1.2, and 4.1.3 reveal that the proposed GSQCCKF algorithm can effectively refine the random model of filter algorithm and mitigate the impact of non-Gaussian noise on the estimation performance in the GNSS/SINS-IADPS under non-Gaussian noise conditions. As a result, the GSQCCKF algorithm exhibits a higher level of computational accuracy and adaptability when compared to algorithms of QEKF, QCKF, and QCCKF. Despite the increased computational time, the GSQCCKF algorithm remains suitable for real-time solution of GNSS/SINS-IADPS under dynamic navigation states.

### 4.2 Experiments and analysis

The performance of GSQCCKF algorithm was evaluated by experiments using UAV that involved a diverse range of maneuvers. The experimental data were collected continuously for a duration of 70 min on September 15, 2023, in Zhengzhou, China (114.0°E, 34.3°N).

The UAV is equipped with a GNSS/SINS-IADPS, the parameters of which are detailed in Table 3. The GNSS reference station is located on the ground, with a maximum distance of 20 km from the UAV. The UAV is also equipped with a GNSS receiver, which processes the paired data between it and the GNSS reference station to obtain the differential GPS (DGPS) data with an accuracy of better than 0.1 m. This DGPS data serves as a reference value for assessing the performance of different algorithms.

**Table 3:** System parameters of GNSS/SINS-IADPS.

Sensors	Parameter	Value
SINS	Gyroscope constant drift	$10^{\circ }/h$
	Random walk coefficient of gyroscope	$0.6^{\circ }/\sqrt{h}$
	Zero deviation of angular velocity meter	40μg
	Random walk coefficient of accelerometer	$80\;\mu g\cdot \sqrt{h}$
	Sampling rate	100/Hz
GNSS	Position measurement noise	15 / m
	Sampling rate	10 / Hz

The starting position of the UAV was 34.654° latitude, 109.193° longitude, and an altitude of 2,683 meters. The initial velocity for the eastern, northern, and up direction are 180 m/s, 60 m/s, and 40 m/s, respectively. Other initial parameters are the same as the simulation. Four different algorithms (QEKF, QCKF, QCCKF, and GSQCCKF) were, respectively, used for GNSS/SINS-IADPS data processing. The test accuracy is measured by 3D positioning error, calculated in formula (43).


(43) $$\Delta p=\sqrt{\Delta \lambda^{2}+\Delta L^{2}+\Delta h^{2}}$$

here, Δλ is the positioning error in longitude; ΔL is the positioning error in latitude; Δh is the positioning error in altitude.

**Figure 8:**
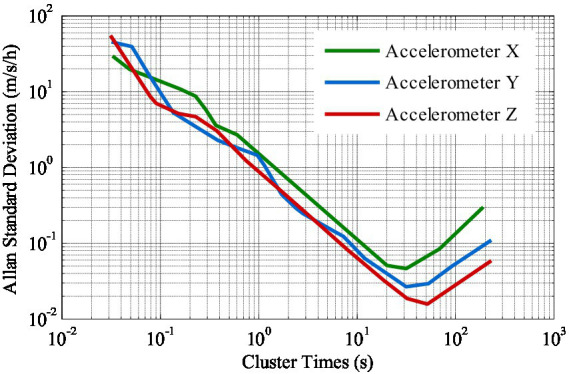
Accelerometer Allan variance results.

To ascertain whether the process noise and measurement noise encountered during experiments are non-Gaussian noise, Allan variance analysis is carried out on the inertial element, the results of which are depicted in Figure 8. The findings indicate that the noise of the inertial element exhibited by the inertial element utilized in the experiments is not white Gaussian noise, but rather a complex noise term encompassing angle random walk, rate slope, quantization noise, and bias instability.

**Figure 9:**
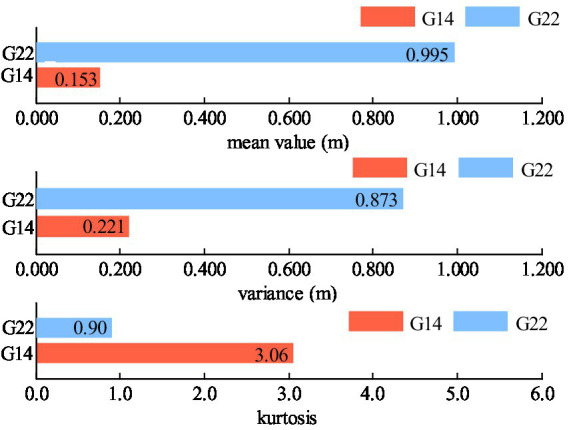
Non-Gaussian characteristics of the pseudo-range noise.

The statistical properties of the pseudo-range noise of satellites with different cutoff angle are shown in Table 4 and Figure 9. Notably, G14 has a higher cutoff angle while G22 possesses a lower cutoff angle. As observed from Table 4 and Figure 9, the kurtosis value of the pseudo noise of the G22 satellite is significantly less than 3, that is, it shows negative kurtosis. Consequently, it can be deduced that the pseudo-range noise of G22 exhibits pronounced non-Gaussian characteristics.

**Table 4:** Statistical characteristics of the pseudo-range noise.

Satellite	Pseudo-range noise		
	Mean value (m)	Variance (m)	Kurtosis
G14	0.153	0.22	3.06
G22	0.995	0.87	0.90

According to Figure 8 and Table 4 as well as Figure 9, the SINS errors and the pseudo-range noise of GNSS present in this experiment data are not white Gaussian noise, but rather non-Gaussian noise.

**Figure 10:**
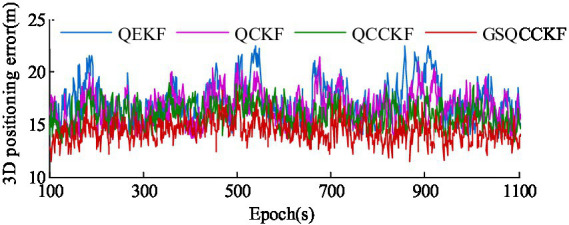
3D positioning errors of UAV for experiments.

**Figure 11:**
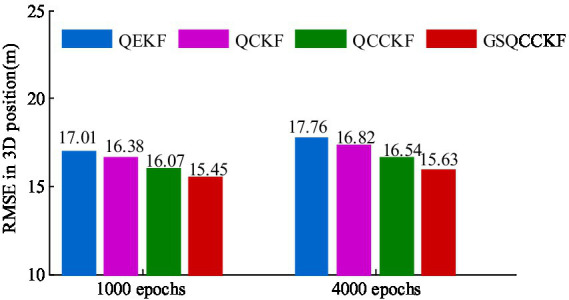
RMSEs of UAV 3D positioning for experiments with different datasets.

The 3D positioning error curves of four different algorithms (QEKF, QCKF, QCCKF, and GSQCCK) for the epoch range ranging from 100s to 1100s are illustrated in Figure 10. The 3D positioning ARMSEs of different algorithms based on 1,000 sets of data and 4,000 sets of data are shown in Figure 11. It is worth noting that the epoch ranges for the 1,000 sets of data encompass the epoch from 100 s to 1100s, while the epoch range for the 4,000 sets of data spans from 100 s to 4,100 s.

As depicted in Figures 10, 11, the period ranges from 100 s to 1100s reveals that the QEKF algorithm is susceptible to linearization errors, resulting in a substantial RMSE of approximately 17.01 m. In contrast, the QCKF algorithm employs a set of cubature points to compute the mean and its covariance, thereby mitigating the linearization error associated with the nonlinear function. Consequently, the QCKF algorithm exhibits a more accurate positioning performance compared to the QEKF algorithm, with the RMSE of approximately 16.38 m. Moreover, because the QCCKF algorithm considers the constraint condition of quaternion, the RMSE is slightly reduced compared with the QCKF algorithm, reaching 16.07 m. Notably, the maximum variation range of the 3D positioning error curves of GSQCCKF algorithm is narrower than that of observed in algorithms of QEKF, QCKF, and QCCKF. This can be attributed to the GSQCCKF algorithm’s use of GMM to accurately modeling the random model, resulting in the smallest RMSE of approximately 15.45 m. Therefore, the same conclusion as the simulation can be obtained, that is, compared with the other three algorithms (QEKF, QCKF, QCCKF), the GSQCCKF algorithm can achieve the best estimation accuracy and adaptability in GNSS/SINS-IADPS data processing.

It can also evident from Figure 11 that, an increase in the experiment data from 1,000 sets to 4,000 sets, the estimation accuracy of four different algorithms (QEKF, QCKF, QCCKF and GSQCCKF) is reduced (QEKF from 17.01to 17.76 m, QCKF from 16.38 to 16.82 m, QCCKF from 16.07 to 16.54 m, and GSQCCKF from 15.45 to 15.63 m). Notably, the estimation accuracy of GSQCCKF algorithm is always better than the other three filter algorithms. When the experiment data comprises 1,000 sets, the 3D positioning RMSEs of GSQCCKF algorithm are about 9.18, 5.545, and 3.70% higher than those of QEKF, QCKF and QCCKF, respectively. As the experiment data increases to 4,000 sets, the accuracy advantage of GSQCCKF algorithm becomes even more pronounced, which is about 11.01, 6.89, and 5.27% higher than those of QEKF, QCKF, and QCCKF, respectively. This shows that the GSQCCKF algorithm possesses robust processing capabilities for non-Gaussian noise and significantly enhances the GNSS/SINS-IADPS estimation accuracy. Furthermore, the GSQCCKF algorithm maintains high accuracy in long-sailing GNSS/SINS-IADPS applications.

## 5 Conclusion

This paper introduces a novel Gaussian sum quaternion constrained cubature Kalman filter algorithm to tackle the limitations of using QCKF algorithm in the non-Gaussian environments for GNSS/SINS-IADPS. The primary contributions of this research are summarized as follows:

1. The framework of GSQCCKF algorithm is set up. Firstly, the QCKF algorithm based on attitude quaternion for nonlinear/Gaussian systems is presented, followed by an analysis of its limitations. Secondly, the idea of GMM is introduced, which employs a set of Gaussian distributions to approximate the PDF of non-Gaussian variables, including process noise, measurement noise, and state estimation. Thirdly, the combination of QCKF algorithm and GMM produces the proposed GSQCCKF algorithm used for nonlinear non-Gaussian system estimation, which essentially consists of a set of parallel QCKFs.
2. In order to address the quaternion normalization problem in attitude estimation of the proposed GSQCCKF algorithm, a two-step projection method is proposed to resolve the quaternion constraint issue. This approach further enhances the accuracy and numerical stability of GSQCCKF algorithm for GNSS/SINS-IADPS data processing.

Results of simulation and experimentation demonstrate that the proposed GSQCCKF algorithm exhibits a remarkable ability to counteract the adverse effects of non-Gaussian noise on state estimation for GNSS/SINS-IADPS, and it demonstrates significantly enhanced estimation accuracy and adaptability in comparison with algorithms of QEKF, QCKF, and QCCKF.

The GSQCCKF algorithm proposed in this article also has limitations. Theoretically, the GSQCCKF algorithm are unable to adjust to time-varying non-Gaussian noise. In practical terms, due to the non-stationary nature of challenging operation environments of GNSS/SINS-IADPS data processing for UAVs, the non-Gaussian noise may vary over time. The inability to effectively mitigate the impact of such time-varying non-Gaussian noise in the GSQCCKF algorithm could potentially distort the performance of GNSS/SINS-IADPS. Future research efforts should focus on discussing modeling approaches for time-varying non-Gaussian noise in GNSS/SINS-IADPS data processing for UAVs.

## Data availability statement

The data analyzed in this study is subject to the following licenses/restrictions: the data that support the findings of this study are available from the corresponding author upon reasonable request. Requests to access these datasets should be directed to QD, daiqing@lypt.edu.cn.

## Author contributions

QD: Funding acquisition, Writing – original draft, Writing – review & editing. G-RX: Methodology, Writing – original draft. G-HZ: Formal analysis, Validation, Writing – review & editing. Q-QY: Visualization, Writing – review & editing. S-YH: Validation, Visualization, Writing – original draft, Writing – review & editing.
